# *Shoshin* Beriberi in Critically-Ill patients: case series

**DOI:** 10.1186/s12937-015-0039-7

**Published:** 2015-05-17

**Authors:** George Dabar, Carine Harmouche, Bassem Habr, Moussa Riachi, Bertrand Jaber

**Affiliations:** 1 Pulmonary and critical section, Hotel Dieu de France Hospital, Saint Joseph University School of Medicine, 16–16830, Beirut, Lebanon; 2 Department of Medicine, Kidney and Dialysis Research Laboratory, Division of Nephrology, St. Elizabeth’s Medical Center, 736 Cambridge Street, 02135 Boston, MA USA

**Keywords:** Fulminant beriberi, Lactic acidosis, Circulatory collapse, Critically ill

## Abstract

Thiamine plays a fundamental role in cellular metabolism. The classical syndrome caused by thiamine deficiency is beriberi, and its fulminant variant, once considered an uncommon finding, is now encountered among the critically ill.

We present a case series of four critically ill non-septic non-alcoholic patients with severe lactic acidosis and refractory cardio-circulatory collapse caused by acute fulminant beriberi, which drastically responded to thiamine administration.

In critical care settings, increased awareness of this life-threatening but reversible condition is a requirement, especially among patients receiving parenteral nutrition and those with unexplained recalcitrant lactic acidosis.

## Background

Thiamine deficiency, also known as *beriberi*, has two major clinical manifestations, dry *beriberi* characterized by neurologic manifestations that include peripheral neuropathy and acute encephalopathy, and wet *beriberi* with cardiovascular involvement including high cardiac output heart failure [1]. Rarely, a fulminant or “pernicious” variant, termed *Shoshin beriberi* may occur, and is characterized by cardiovascular collapse. Appropriate management of this form is mandatory since thiamine supplementation leads to rapid recovery while untreated forms are fatal [2]. Complex clinical presentations in patients without a history of alcohol abuse or dependence or overt malnutrition and the lack of a high index of suspicion coupled to lack of rapid diagnostic testing in emergency settings, make thiamine deficiency a potentially missed diagnosis [3].

**Fig. 1:**
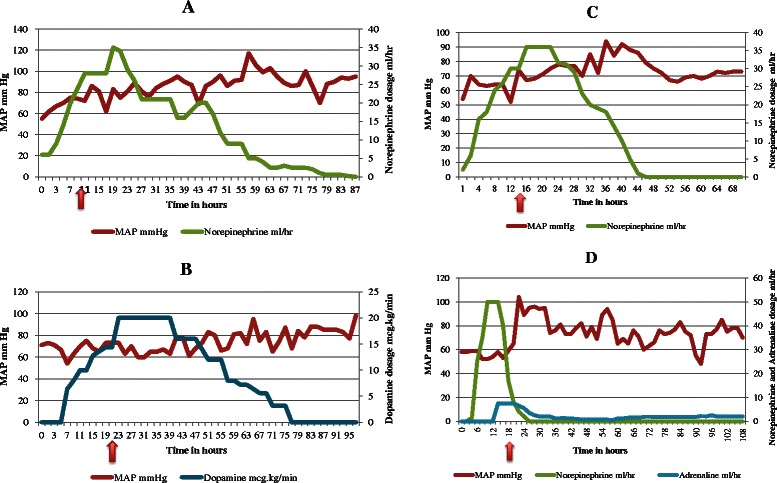
The four charts represent the trend of MAP along with the vasopressor doses prior and following Thiamine infusion. In the four charts the x- Axis is hours and the Arrow points at the time of the initial Thiamine infusion.1.**a**. Case # 1. 1.**b**. Case # 2, 1.**c**.: Case # 3, 1.**d**.: Case # 4. In the charts Epinephrine (Adrenaline) and Norepinephrine are shown in milliliters per hour, conversion to mg per hour is 0.25 mg/ml and 0.2 mg/ml respectively

In this article, we present 4 cases (Table 1) of hospitalized patients without history of alcohol dependence, encountered between 2008 and 2009, who suffered from recalcitrant metabolic acidosis and refractory hypotension (Fig. 1) caused by acute fulminant cardiac beriberi, and a review of the literature is done.

**Table 1:** Characteristics and outcomes of the 4 critically ill patients with presumed *Shoshin* beriberi

Case No.	Underlying Disease	Duration of total parenteral nutrition	Nadir serum bicarbonate (mmol/L)	Peak lactate (mmol/L)	Initial thiamine intravenous dosing regimen	Time to hemodynamic recovery	Outcome
1	Gastric surgery	15 days	9	23	100 mg daily for 50 days	48 hours	Recovery
2	Pancreatic cancer	29 days	11	14	100 mg daily for 26 days	6 hours	Recovery
3	Type-1 glycogen storage disease	-	8	32	100 mg daily for 25 days	24 hours	Recovery
4	Peritonitis	26 days	9	35	100 mg daily for 13 days	12 hours	Death

### Case #1

A 45 year-old man was transferred from the medical ward to our intensive care unit (ICU) for evaluation and management of acute abdominal pain, severe hypotension unresponsive to fluids and severe lactic acidosis. He had a history of gastric resection with a Roux-en-Y anastomosis due to peptic ulcer disease performed fifteen years ago. He presented to the hospital 15 days earlier with recurrent vomiting deemed to be secondary to dumping syndrome. Oral intake was interrupted and total parenteral nutrition (TPN) was initiated without water-soluble vitamin supplementation. There was no known history of alcoholism or liver disease. Upon transfer to the ICU, he was intubated and placed on mechanical ventilation. Refractory hypotension required vasopressor support. The arterial blood gas revealed severe metabolic acidosis with a pH of 7.02, PaCO2 of 19 mmHg, PaO2 of 182 mmHg, and bicarbonate level of 9 mmol/L (normal range 24–28). Serum lactate level was markedly elevated at 23 mmol/L (normal range 0.5–2.2). No infectious source was identified despite total body scan imaging studies. He deteriorated and became hemodynamically unstable requiring higher doses of vasopressors. In the presence of unexplained lactic acidosis and the recent exclusive use of TPN without water-soluble vitamin supplementation, thiamine deficiency was suspected. Thiamine was immediately administered intravenously (100 mg) followed by a daily intravenous infusion of 100 mg for the remaining hospital stay. Vasopressors were weaned off 48 h following the first thiamine dose. The patient was successfully extubated on day 4 was transferred to regular floor on day five and was discharged home 45 days later.

### Case #2

A 60 year-old woman with a diagnosis of pancreatic adenocarcinoma associated with peritoneal metastases presented to the emergency room for nausea and vomiting. Ileal occlusion by extrinsic tumor compression was diagnosed. A gastro-jejunal anastomosis was performed and the patient was initiated on TPN with no water-soluble vitamins. Her hospital course was complicated by a nosocomial pneumonia treated appropriately. On the 37^th^ hospital day, the patient hemodynamic status deteriorated, and she was transferred to the ICU. Persistent hypotension despite adequate fluid resuscitation required use of vasopressors. The laboratory investigations were inconclusive except for pronounced lactic acidosis with a pH of 7.38, a pCO2 of 21 mmHg, a lactate level of 13.6 mmol/L, and bicarbonate level of 11 mmol/L. Liver and kidney function tests were normal. A CT-scan of the chest, abdomen and pelvis did not reveal any source of sepsis except for the resolving pulmonary consolidation. Her hemodynamic instability necessitated escalating doses of vasopressors. Exclusive TPN infusion for 21 days in conjunction with unexplained lactic acidosis and persistent shock were highly suggestive of wet beriberi. After a single dose of intravenous thiamine (100 mg) the mean arterial pressure improved within minutes, and the patient was able to be weaned off vasopressors in the following hours as her hemodynamic instability resolved. She was kept on a daily dose of 100 mg of Thiamine. Serum lactate level returned to normal values within 24 h. In the following days, enteral nutrition was initiated and she was discharged from the ICU. The patient was discharged home 21 days later.

### Case #3

A 23 year-old man was admitted to our hospital with a 2-week history of nausea and vomiting, generalized weakness, severe loss of appetite and paraparesis. He had a known diagnosis of Von Gierke’s disease or type-1 glycogen storage disease, supplemented by starches since childhood. He used no medications, and had no known history of alcohol abuse or liver dysfunction. Laboratory studies and neurological investigations, including imaging studies and a lumbar puncture, were unrevealing. On his second day of hospitalization, he became drowsy and hypotensive. Pronounced lactic acidosis (31 mmol/L) was identified with an arterial pH of 7.19, pCO2 of 8 mmHg, pO2 of 140 mmHg, and bicarbonate level of 7.7 mmol/L. Acute kidney injury secondary to severe hypotension with acute tubular necrosis complicated his course and necessitated renal replacement therapy transiently. He had no evidence of infection. He was transferred to our ICU, where he was intubated and placed on mechanical ventilation. He remained hemodynamically unstable despite escalating doses of vasopressors. Severe lactic acidosis associated with circulatory collapse and neurological dysfunction suggested thiamine deficiency. Thiamine (100 mg) was immediately administered intravenously. Lactic acidosis and body temperature improved during the first 12 h followed by the weaning of vasopressors and an improvement in the urine output within 24 h. Multiple organ failure resolved within two days. He received a daily dose of 100 mg of thiamine for the remaining hospital days. He was transferred to regular floor on day 25, and was eventually discharged home.

### Case #4

A 48-year old woman was admitted to the ICU with severe hypotension and lactic acidosis, as evidenced by a pH of 7.27, pCO2 of 21 mmHg, pO2 of 70 mmHg, bicarbonate of 9 mmol/L and lactate level of 5 mmol/L. The patient had chronic kidney failure on maintenance peritoneal dialysis (PD). She had been recovering from an episode of PD catheter-related peritonitis diagnosed a month earlier, and had been receiving TPN not supplemented with water-soluble vitamins on the medical ward for 26 days. At the time of admission, she was confused and required intubation with mechanical ventilation. Infectious sources for potential sepsis could not be identified. Vasopressor requirement increased significantly, with a lactate level reaching a peak of 34 mmol/L with persistent metabolic acidosis. The patient’s hemodynamic status failed to improve. On the basis of the patient’s history of chronic use of TPN, and persistent lactic acidosis and cardiovascular collapse in the absence of other causes of shock, thiamine deficiency was suspected, and she received a 200-mg loading dose of thiamine. Within a few hours, the lactic acidosis resolved and the vasopressors were weaned off. She was maintained empirically on high-dose thiamine substitution (100 mg twice daily) for a planned duration of 2 weeks. In the following days, although the patient stabilized hemodynamically, she subsequently developed small bowel ischemia, which was further complicated by disseminated intravascular coagulation leading to death.

## Discussion

Thiamine (or vitamin B1) deficiency, also known as *beriberi*, has traditionally been divided into two major types: a “dry” form, in which features of peripheral neuropathy predominate, and a “wet” form, in which signs and symptoms of right-sided heart failure with normal or high cardiac output are the presenting features. A fulminant variant, termed *Shoshin beriberi* (from the Japanese *sho* meaning acute damage, and *shin* meaning heart), complicating either one of the two types, may occur with severe biventricular failure, metabolic acidosis, variable cardiac output with vascular collapse, peripheral cyanosis and eventually death [4]. This syndrome is usually preceded by non-specific symptoms such as generalized fatigue, loss of appetite, and abdominal pain.

**Fig. 2:**
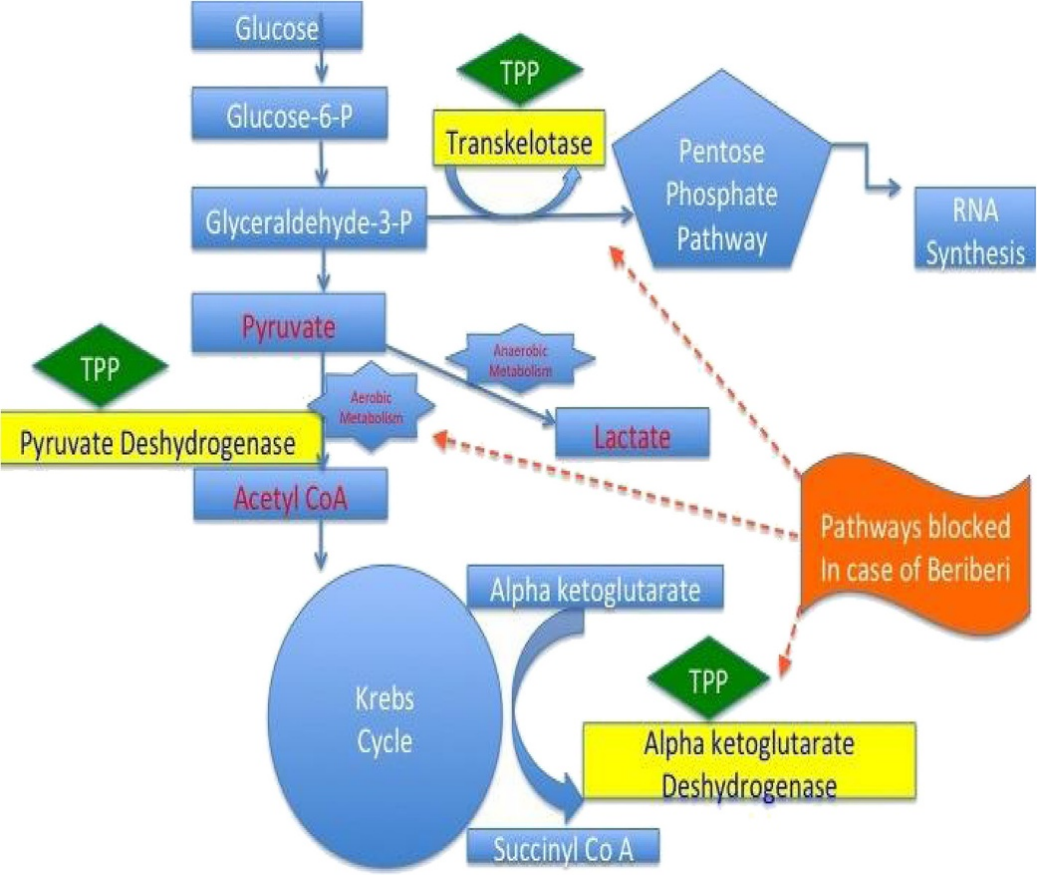
Carbohydrate metabolism and role of thiamine. The inability to use the Krebs cycle is the major underlying pathophysiological feature of thiamine deficiency. Thiamine pyrophosphate (TPP), is an essential component of aerobic metabolism. A decrease in its activity may lead to the tissue accumulation of toxic intermediates such as pyruvate and lactate. TPP: Thiamine Pyrophosphate, Glucose 6-P: Glucose 6 Phosphate, Glyceraldehyde 3 P: D-Glyceraldehyde 3 Phosphate, RNA Synthesis: Ribonucleic Acid Synthesis, Acetyl CoA: Acetyl Co-enzyme A, Succinyl CoA: Succinyl Co-enzyme A

Acidosis and the inability to utilize the Krebs cycle are the major pathophysiological underpinnings of the clinical manifestations of thiamine deficiency [5]. Thiamine, in its phosphorylated form, thiamine pyrophosphate (TPP), is an essential component of aerobic metabolism [6, 7]. It is the precursor for the co-factor of both pyruvate dehydrogenase and alpha-ketoglutarate dehydrogenase: two enzymes that catalyze the oxidative decarboxylation of pyruvate to acetyl-CoA and alpha-ketoglutarate to succinyl-CoA, respectively [7]. Pyruvate dehydrogenase and alpha-ketoglutarate dehydrogenase are both key enzymes of the Krebs cycle (Fig. 2). A decrease in their activity may lead to the tissue accumulation of toxic intermediates such as pyruvate and lactate. Symptoms of lactic acidosis and organ dysfunction will eventually occur. Thiamine is also a coenzyme in the utilization of the pentose phosphate pathway, which serves as an alternative pathway for glucose oxidation as well as an important route for ribose nucleic acid synthesis (Fig. 2).

In developed countries, thiamine deficiency occurs mainly in persons with alcohol abuse and dependence, food faddists, and in the elderly [8]. It should be suspected in patients with severe sepsis-like syndrome, unexplained heart failure or lactic acidosis, unexplained neurological disorders in the setting of alcoholism, starvation, chronic malnutrition, total parenteral nutrition, hyperemesis gravidarum, or following surgery [9, 10].

A prospective observational study of critically ill adults admitted to the ICU with the diagnosis of severe sepsis and septic shock found a relatively high prevalence rate (35.7 %) of thiamine depletion [8]. A potential relationship between thiamine levels and lactic acidosis has been demonstrated in critically ill septic patients [11]. Thiamine levels have also been found to be low in critically ill children [12] and in other clinical settings, including acute leukemia, cardiac and bariatric surgery, and burns [9, 10].

Acute fulminant *beriberi*, previously considered as an uncommonly encountered clinical entity [4], is not a rare occurrence anymore in the ICU. Many isolated cases have been described [4, 7, 13–15]. Two previous large case series collected a significant number of cases, among a South African population of patients with alcoholism [16] and in an endemic island of the Indian Ocean afflicted by food shortage [17]. Three of our four cases of *Shoshin* beriberi encountered over an 18-month period in critically ill patients without a history of alcoholism had the common feature of TPN use. One prior report in a similar context identified ten patients with TPN-induced fulminant beriberi encountered over an 8-year period [5]. In our case series the patients received a three in one TPN-formula by non-critical care physicians unaware of lack of vitamin supplementation. A summary of the previously published cases of *Shoshin* Beriberi is provided in Table 2. Patients with Von Gierke’s disease are prone to lactic acidosis as a result of hepatic glucose-6-phosphate deficiency leading to diversion to glycolysis and the production of excess pyruvate, at levels above of the capacity of the Krebs cycle to completely oxidize. This results in pyruvate reduction to lactate and development of lactic acidosis. The underlying mechanism for development of thiamine deficiency is unknown. We can only speculate as to whether patients with type-1 glycogen storage disease fed on a diet rich in starch and glucose tend to utilize thiamine in the form of thiamine pyrophosphate, and when in severe lactic acidosis, thiamine supplementation might facilitate the conversion of pyruvate to acetyl-coA thus entering the Krebs cycle and attenuating lactate production.

**Table 2:** Summary of previously published case series of *Shoshin* Beriberi

Author [ref]	Year	No. patients with *Shoshin* beriberi	Thiamine supplementation	Thiamine initial dose	Outcome
Pereira et al. [19]	1984	2	Yes	Not specified	Recovery (2)
Naidoo [16]	1987	8	Yes	Not specified	Recovery (8)
Kitamura et al. [5]	1996	10	Yes (6)	10 mg (1)>	Death (1)
				100 mg (5)	Recovery
					(5) Death (4)
Shivalkar et al. [20]	1998	2	Yes	500 mg (2)	Recovery (2)
Bello et al. [7]	2011	2	No	Not specified	Death (2)
Restier et al. [17]	2012	11	Yes, early (8)	Not specified	Recovery
					(8) Death (3)

The diagnosis of *Shoshin* beriberi is often difficult to entertain in a critical care setting due to its protean clinical manifestations, the low index of suspicion, and the lack of readily available emergent blood thiamine measurements [7]. Measurements of blood thiamine concentration or of the red blood cell transketolase activity lack specificity and are not widely available laboratory tests [18]. Blood measurements were unfortunately not performed in our patients due to the non-availability of these assays. Another limitation is the absence of echocardiography documentation prior to thiamine supplementation, which would have been helpful but not necessary for the diagnosis of biventricular failure secondary to thiamine deficiency [4].

The only definitive treatment of beriberi is a therapeutic trial of rapid intravenous administration of thiamine, which improves hemodynamic parameters within minutes to hours; in the setting of a high index of suspicion for presumed thiamine deficiency, this emergent approach is the only way to rapidly diagnose suspected beriberi. In our four patients, thiamine administered intravenously promptly reversed both the profound cardiovascular collapse and metabolic disturbances within 6 to 48 h (Fig. 1).

## Conclusions

We report a case series of presumed fulminant beriberi in four critically ill patients, three had received TPN without water-soluble vitamin supplementation and one had type-1 glycogen storage disease. Since thiamine is not routinely administered to critically ill patients, these observations emphasize the necessity, in an ICU setting, of maintaining a high index of suspicion for this life-threatening but reversible diagnosis, especially among patients receiving TPN, or those with unexplained recalcitrant lactic acidosis, and embarking on an emergent empiric therapeutic trial of thiamine supplementation to rapidly cure a potential hemodynamic disaster [2].

## Consent

Institutional ethics committee waived informed patient consent due the observational nature of the study and in concordance with the current applicable laws.
